# Audit examining memantine initiation in dementia patients in an older adult service in the north west

**DOI:** 10.1192/bjo.2021.225

**Published:** 2021-06-18

**Authors:** Irina Casapu, Ste Dickinson, Chirag Shroff, Sofia Almeida, Kieran McSharry

**Affiliations:** 1 CT3, Liaison Psychiatry, Aintree Hospital; 2 F3, A and E, Royal Liverpool University Hospital; 3 CT2, Burlington House CAMHS, Alderhey NHS Trust; 4 CT3, Liaison Psychiatry, Countess of Chester Hospital; 5 ST5 Dual Old Age and General Adult Trainee, Merseycare NHS

## Abstract

**Aims:**

Dementia is a progressive condition inflicting significant costs for health and social care services. In December 2017, there were 456,739 people on GP registers with a formal diagnosis of dementia. Making the right choice of anti-dementia medication with essential monitoring is one important aspect of care. Thus, the aim of this audit was to identify if current practice at Mossley Hill inpatients and outpatients service for older adults in Liverpool, was in accordance with the NICE Guideline NG97 (Dementia: assessment, management and support for people living with dementia and their carers). Additionally, we aimed to evaluate whether Memantine was commenced according to BNF/SPC recommendations about e-GFR and whether this was documented on patient records, as well as to highlight areas of improvement.

**Method:**

An audit was carried out for all patients for whom Memantine was initiated, between June and August 2019. Sixty-nine patients were identified through trust Pharmacy records. Data were collected retrospectively, reviewing local electronic records (ePEX, RIO) and GP referrals. This included age, sex, diagnosis, indication for starting Memantine, decision context, prescriber, documentation of renal function status and communication of decision to the GP. Findings were compared to NICE guidance NG97 and presented at the local audit meeting with a view to recommend strategies for improvement.

**Result:**

Results indicated that most of the patients were female (64%) with the most common diagnosis being Alzheimer's disease (75%). Recurrent reasons for initiating Memantine were: contraindication for AChE treatment (25%); illness progression on AChE (22%); and severe dementia on initial presentation (23%). Usually, the decision to start Memantine treatment was made in MDT or after prescriber clinical review. In 68% of the reviewed cases, renal function status was documented. Patients' GP was informed of medication change in 86% of cases.

**Conclusion:**

To conclude, in the majority of cases Memantine initiation was in line with NICE guidance. However, documentation can be improved, so as to facilitate future audit. We recommended creating a checklist for prescribing Memantine that could be integrated within the electronic records system.

